# Effect of a mobile-based, family-centered self-care education program on Health Literacy and Self-Care in Patients with Heart Failure: A Randomized Controlled Trial

**DOI:** 10.17533/udea.iee.v43n3e09

**Published:** 2025-10-28

**Authors:** Zohreh Badiyepeymaiejahromi, Mahsa Khorramkish, Ali Asghar Rahmaniankushkaki

**Affiliations:** 1 Faculty member, Ph.D. Assistant Professor. Email: z.badiyepeyma@gmail.com https://orcid.org/0000-0001-6643-036X Jahrom University of Medical Sciences Iran z.badiyepeyma@gmail.com; 2 Nurse, M.Sc. Email: Khoramkish68@gmail.com https://orcid.org/00000-0003-3571-1470 Jahrom University of Medical Sciences Iran Khoramkish68@gmail.com; 4 Department of Medical- Surgical Nursing, Jahrom University of Medical Sciences, Jahrom, Iran. https://orcid.org/0000-0001-9516-833X Jahrom University of Medical Sciences Department of Medical- Surgical Nursing Jahrom University of Medical Sciences Jahrom Iran

**Keywords:** self-care, family-centered, heart failure, randomized controlled trial, health literacy, cell phone, health education, cell phone, mobile applications., autocuidado, cuidado centrado na família, insuficiência cardíaca, ensaio clínico controlado aleatório, letramento em saúde, telefone cellular, educação em saúde, aplicativos móveis, autocuidado, cuidado centrado en la familia, insuficiencia cardíaca, ensayo clínico controlado aleatorio, alfabetización en salud, teléfono celular, educación en salud, aplicaciones móviles.

## Abstract

**Objective.:**

To evaluate the effect of family-centered self-care education via tele-nursing on health literacy and the self-care status of patients with heart failure.

**Methods.:**

This study is a randomized, controlled clinical trial. Sixty heart failure patients were randomly allocated into two groups: intervention (*n*=30) y control (*n*=30). The researcher conducted six 20-minute sessions on how to take care of yourself in heart failure for active family members of patients in the intervention group via mobile phone on Eitaa APP messenger. Before the intervention and one month after it, patients completed the Heart Failure Health Literacy Questionnaire and the Self-Care Assessment Questionnaire.

**Results.:**

Inter-group comparisons using the Mann-Whitney test showed that before the intervention, there was no statistically significant difference between the intervention and control groups in terms of mean health literacy scores and self-care behaviors(*p*<0.05). However, after the intervention, a significant difference emerged, with the intervention group exhibiting higher mean scores than the control group in both questionnaires (*p*<0.001). Intra-group comparisons further revealed that the intervention group’s mean scores for health literacy and self-care behaviors increased significantly after the intervention compared to before (*p*<0.001).

**Conclusion.:**

Family-centered education via mobile phone on Eitaa APP messenger improved both health literacy and self-care status among heart failure patients. Thus, employing tele-nursing to engage the family members of heart failure patients can be an effective strategy for delivering educational interventions as part of the treatment program.

## Resumen

**Objetivo.:**

Evaluar el efecto de una intervención educative de enfermería, centrada en la familia, basada en el uso de dispositivos móviles en el mejoramiento de los conocimientos de salud y del estado de autocuidado de los pacientes con insuficiencia cardíaca.

**Métodos.:**

Este estudio es un ensayo controlado aleatorizado. Se asignaron sesenta pacientes con insuficiencia cardíaca a dos grupos: intervención (*n*=30) y control (*n*=30). El investigador llevó a cabo seis sesiones educativas de veinte minutos sobre el autocuidado a los familiares de las personas con insuficiencia cardíaca del grupo de intervención, utilizando en los teléfonos móviles la aplicación de mensajería Eitaa. Antes y un mes después de la intervención, los pacientes completaron el Cuestionario de Alfabetización Sanitaria sobre Insuficiencia Cardíaca y el Cuestionario de Evaluación del Autocuidado.

**Resultados.:**

Las comparaciones entre grupos mostraron que, antes de la intervención, no habían diferencias estadísticamente significativas en cuanto a las puntuaciones medias de conocimientos sobre salud y en los comportamientos de autocuidado (*p*<0.05). Sin embargo, después de la intervención, se observó que el grupo de intervención obtuvo puntuaciones medias más altas que el grupo de control en ambos cuestionarios (*p*<0.001). Las comparaciones intragrupales confirmaron, además, que las puntuaciones medias del grupo de intervención en cuanto a conocimientos sobre salud y comportamientos de autocuidado aumentaron significativamente después de la intervención en comparación con la evaluación basal (*p*<0.001).

**Conclusión.:**

El programa de educación de enfermería centrada en la familia basado en el uso del teléfono móvil con la aplicación de mensajería Eitaa mejoró tanto la alfabetización en salud como el estado de autocuidado entre los pacientes con insuficiencia cardíaca. Por lo tanto, el uso de intervención que involucra a los familiares de los pacientes con esta enfermedad puede ser una estrategia eficaz para llevar a cabo el componente educativo como parte del programa del tratamiento.

## Resumo

**Objetivo.:**

Avaliar o efeito de uma intervenção de educação em enfermagem centrada na família e baseada em dispositivos móveis na melhoria da alfabetização em saúde e do autocuidado em pacientes com insuficiência cardíaca.

**Métodos.:**

Este estudo é um ensaio clínico randomizado. Sessenta pacientes com insuficiência cardíaca foram divididos em dois grupos: intervenção (n = 30) e controle (n = 30). O pesquisador conduziu seis sessões educativas de autocuidado, com duração de vinte minutos, com familiares de pessoas com insuficiência cardíaca no grupo intervenção, utilizando o aplicativo de mensagens Eitaa em seus celulares. Antes e um mês após a intervenção, os pacientes responderam ao Questionário de Alfabetização em Saúde para Insuficiência Cardíaca e ao Questionário de Avaliação de Autocuidado.

**Resultados.:**

As comparações entre os grupos mostraram que, antes da intervenção, não houve diferenças estatisticamente significativas nas médias de alfabetização em saúde e comportamentos de autocuidado (*p*<0.05). No entanto, após a intervenção, o grupo de intervenção obteve pontuações médias mais altas do que o grupo controle em ambos os questionários (*p*<0.001). As comparações intragrupo também confirmaram que as pontuações médias do grupo de intervenção em alfabetização em saúde e comportamentos de autocuidado aumentaram significativamente após a intervenção em comparação com a avaliação inicial (*p*<0.001).

**Conclusão.:**

O programa de educação em enfermagem centrado na família, baseado no uso de telefones celulares com o aplicativo de mensagens Eitaa, melhorou tanto a alfabetização em saúde quanto o status de autocuidado entre pacientes com insuficiência cardíaca. Portanto, o uso de uma intervenção envolvendo familiares de pacientes com insuficiência cardíaca pode ser uma estratégia eficaz para implementar o componente educacional como parte do programa de tratamento.

## Introduction

Heart failure (HF) has been defined as a global pandemic, with 64.3 million people estimated to suffer from HF worldwide in 2017.^1^ The 2021 American Heart Association Heart Disease and Stroke Statistics based their HF prevalence estimates on the NHANES data collected between 2015 and 2018. Around 6.0 million Americans aged ≥20 years had HF, which increased from around 5.7 million according to National Health and Nutrition Examination Survey data collected between 2009 and 2012. The prevalence of HF in the USA was 2.4% in 2012, which is projected to rise to 3.0% in 2030.^2^ At least 50% of heart failure patients do not follow the treatment recommendations related to dietary regimens and medication instructions, which leads to their re hospitalization.^3^ According to recent heart failure guidelines, it can be concluded that patients with heart failure need to have knowledge about their condition, including both pharmacological and non-pharmacological treatments, as well as the ability to recognize symptoms and understand what may worsen them.^4^ The results of Bagheri Saveh *et al.*’ study indicated that the self-care status of patients with heart failure is moderate.^5^The effects of self-care behaviors in people with heart failure are equivalent to drug regimens. In people with heart failure, optimal self-care can lead to maintaining physical health, preventing disease exacerbations, improving clinical outcomes, reducing mortality and morbidity, reducing hospitalizations, and improving quality of life.^6^^-^^8^

One of the factors influencing patient adherence to treatment recommendations is health literacy.^9^ Health literacy is defined as the extent of individuals’ capacity to acquire, interpret, and understand the health information and services necessary for making appropriate decisions.^10^ Low health literacy is not only a fundamental issue for patients, but also for healthcare providers and health systems.^11^ In the study by Marzangi *et al*., it was found that 85% of heart failure patients lacked sufficient information regarding the nature of the disease, 92.5% lacked adequate information about the dietary regimen, 95% lacked sufficient information regarding the medication regimen, 82.5% lacked the necessary information about rest and sexual activity, and 85% lacked adequate information regarding treatment follow-up.^12^

Education for family members in the control and even prevention of disease can be extremely beneficial, given the strong connection between the family and the health status of its members. Individuals, especially those with chronic illnesses, rely on their family members, and even their attitudes are influenced by their family. In family-centered education, the family actively participates in identifying needs and providing the necessary training for patients, as it is believed that the onset of an illness in an individual involves each family member in the patient’s overall disease process.^13^ Today, the concept of patient and family empowerment has taken on a special significance. By operationalizing this concept and involving family members of patients, it is possible to help them take effective steps toward quicker problem resolution and improvement of the patient’s condition. This is achieved by increasing their knowledge of care, enhancing their understanding of the disease, and reducing both physical and psychological stresses.^14^^,^^15^ Educating patient caregivers and providing them with sufficient and useful information will lead to improved recovery and better adaptation to the disease for both the patient and their family.^16^ This educational approach, in addition to being applied in HF,^17^ has also been implemented in other conditions, including type 2 diabetes^18^ and multiple sclerosis^19^ and has demonstrated a positive impact.

Today, with the increasing spread of mobile internet, smartphones, and portable personal applications, remote healthcare has become accessible. Remote healthcare or virtual health education encompasses educational programs, motivating adherence to self-care patterns, and assisting in the regular monitoring of symptoms. Digital technology and multimedia environments are among the tools that can aid in enhancing patients’ self-care. Important features of virtual education include easy access, flexibility, the elimination of costly travel, and the ability to revisit the content.^20^ Mobile phone education services can be provided for all age groups, from newborns to the elderly. One of the key benefits of mobile phone education programs is the reduced risk of exposure to infections for both patients and healthcare personnel while ensuring safe care and treatment. Additionally, mobile phone education offers an opportunity to deliver higher-quality care and expand access to nursing services. Through mobile phone education, nurses can remotely monitor, educate, follow up, collect data, provide interventions, manage pain, support families, and deliver multidisciplinary care in an innovative manner, ultimately reducing healthcare and treatment costs for patients.^21^ Family-centered self-care education through mobile phone education has been implemented in patients with COVID-19,^22^ type 2 diabetes,^23^ and heart attack,^24^ showing positive effects. Barkhordari *et al.* also demonstrated the positive impact of virtual health literacy education on the self-care behaviors of heart failure patients.^25^

 Although the effectiveness of family-centered self-care education through mobile phone has been proven in the aforementioned studies, it cannot be definitively concluded that this method of education has a positive or negative effect on patients with heart failure, Because the nature of this disease is different from other diseases, and therefore the self-care education that the patient and family must receive is also different, it is necessary to conduct this study to draw the correct conclusion. Considering the above, the purpose of the present study is effect of mobile-based, family-centered self-care education program on Health Literacy and Self-Care in Patients with Heart Failure.

## Methods

Study Design. This study is a randomized controlled clinical trial, which has been adhered the Consolidated Standards of Reporting Trials (CONSORT) Guidelines.^26^ The study population includes all patients with heart failure who visited a cardiologist at the Imam Reza Clinic, affiliated with Jahrom University of Medical Sciences, located in southern Iran. To estimate the sample size based on the health literacy variable, a study by Kobraei *et al.*^27^ was used. Considering a Type I error of 5%, an effect size of 0.801, and a power of 80%, the sample size for each group was calculated as 26 participants. Accounting for a 10% dropout rate, the final sample size in each group was determined to be 30 participants, resulting in a total of 60 participants. The sample size was calculated using the formula below with G*Power software.




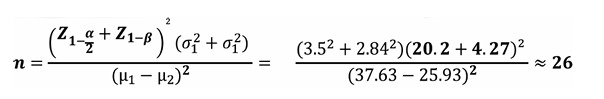




Sampling and Randomization. Sampling was conducted using a convenience sampling method at Imam Reza Clinic, affiliated with Jahrom University of Medical Sciences. The process began in October 2023 to March 2024. Patients were gradually assessed and selected by the principal investigator based on predefined inclusion criteria during their routine visits to the cardiologist. Eligible participants were then randomly assigned to either the intervention or control group using simple randomization via computer software (Random Allocation Software). The randomization sequence was generated prior to recruitment by an independent statistician using the same software. Allocation concealment was ensured through the use of opaque, sealed, and sequentially numbered envelopes, which were opened only after participant enrollment. Participant enrollment and group assignment were carried out by a research assistant who was blinded to the randomization sequence and was not involved in outcome assessment, thereby minimizing potential allocation bias.

Inclusion and Exclusion Criteria. (i) Patient Inclusion Criteria: Diagnosis of heart failure confirmed by the cardiologist, Classification as NYHA (New York Heart Association) class II or III, Age between 18 and 65 years, having active family members with accessible contact, Willingness to participate in the study; (ii) Patient Exclusion Criteria: Presence of psychological disorders based on self-report,Worsening of the disease condition, Death of the patient; (iii) Family Member Inclusion Criteria: Being an active caregiver for the patient as confirmed by the patient, Age 18 years or older, Basic literacy (ability to read and write), Owning a smartphone with the Eitaa app installed, Willingness to participate in the study; and (iv) Family Member Exclusion Criteria: Presence of psychological disorders, Missing more than two educational sessions.

Blinding. Given that the intervention was implemented by the researchers themselves and the sampling was conducted at a single center, it was not feasible to blind either the researchers or the participants. However, the individual responsible for data analysis remained blinded to group allocation throughout the study. Although the outcome assessor was the principal investigator and blinding was not implemented during data collection, all outcomes were measured using validated and standardized questionnaires. All questionnaires were completed through telephone interviews conducted by the researcher, following standardized instructions to ensure consistency across all participants and minimize potential bias in data collection. This approach helped reduce subjective interpretation and thereby limited the risk of bias in outcome assessment.

Intervention. The study received the code of IRCT20210114050037N2 in the Clinical Trial Registration Center of Iran. After obtaining informed consent, the relevant questionnaires were completed via telephone by patients in both the intervention and control groups. In the intervention group, patients were asked to identify an active family member who played the most significant role in their care and could participate in the educational program. Once the patient selected an active family member, their contact information was obtained, and the researcher coordinated with them, explaining the study details. It is important to note that a patient’s family member could be their child, spouse, or a relative (either by blood or marriage) who actively supports the patient’s recovery and overall health improvement.^28^ The selected active family member was expected to be capable of providing support, making decisions, and understanding the patient’s condition better than others. After completing the initial questionnaires, a six-session educational program on self-care for heart failure patients was delivered to the active family member of the intervention group via the Eitaa app. 

A dedicated group was created on Eitaa, where all active family members in the intervention group were added. Educational content was shared in the form of short instructional videos, uploaded on a designated day each week. Active family members were given time to watch the videos. One to two days after each session, the researcher followed up with active family members via phone to confirm that they had watched the videos, received appropriate feedback, and conveyed the information to the patient. Each session lasted approximately 20 minutes. Throughout the program, the Eitaa group remained active, allowing caregivers to ask questions and engage in discussions. During the intervention, both the control and intervention group patients continued their routine medical visits and received standard care and education provided by their physician, without any additional training.

Educational Session Topics: Session 1: Understanding heart failure and its treatment options, Session 2: Medication management and adherence in heart failure patients, Sessions 3 & 4: Nutrition for heart failure patients, Session 5: Physical activity for heart failure patients, Session 6: Mental health in heart failure patients.

**Figure 1:**
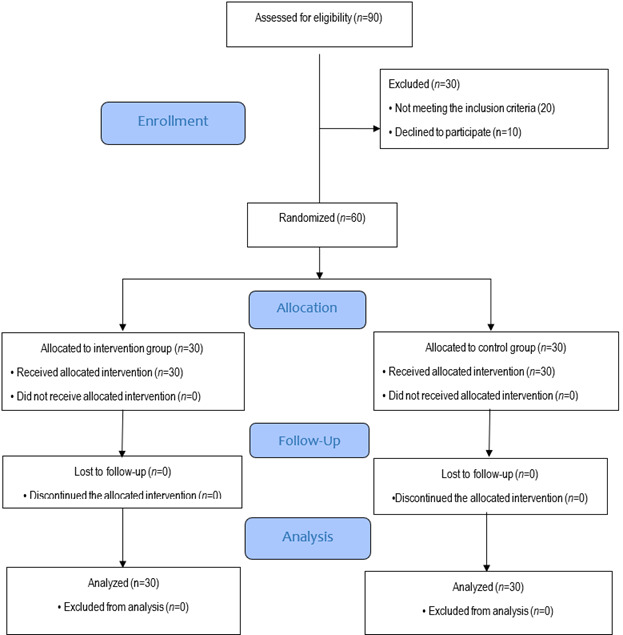
CONSORT Diagram

One month after the intervention, the questionnaires were re-administered via telephone. The researcher contacted each patient, read the questionnaire items, and recorded their responses (Figure 1). Fortunately, no participants dropped out during the study. As a result, the final sample size exceeded the minimum required, which enhanced the statistical power and precision of our findings. Although a one-month follow-up period may be considered relatively short for evaluating long-term behavior change, this timeframe was selected based on several practical and methodological considerations. First, it allows for the assessment of immediate post-intervention effects, providing insight into early behavioral responses and short-term adherence. Second, a shorter follow-up enhances participant retention and reduces the risk of attrition, which is particularly important in pilot studies or when working with vulnerable populations. Third, logistical and resource constraints necessitated a concise follow-up window to ensure feasibility within the scope of the current study.

Nevertheless, the importance of long-term evaluation is acknowledged. Accordingly, extended follow-up assessments (e.g., at 3 or 6 months) are planned to examine the sustainability of behavioral changes over time and to identify potential relapse or adaptation patterns. This limitation was acknowledged in the discussion section, where the relatively short one-month follow-up period was noted as a constraint in evaluating long-term behavioral outcomes.

Data Collection Tools. Data were collected using the following instruments: (*i) Demographic Information Form*: This form included variables such as age, gender, marital status, education level, occupation, disease duration, place of residence, number of hospitalizations, and history of underlying diseases; *(ii) Heart Failure Health Literacy Questionnaire (HFHLQ)*: This questionnaire consists of 12 items measuring three subscales of health literacy in heart failure patients: Functional health literacy (4 items), Communicative health literacy (4 items), Critical health literacy (4 items). The items are rated on a 4-point Likert scale (1 = not at all, to 4 = very much), with a total score ranging from 12 to 48. Some items are reverse-scored. Higher scores indicate better health literacy. The validity and reliability of the original version of this questionnaire have been confirmed.^29^ In Iran, Farghadani *et al.* validated the Persian version through a translation-back translation process by two independent bilingual translators. Content validity was assessed by 10 experts, with recommended modifications applied. The content validity index for clarity, relevance, and simplicity was between 0.8 and 1.0. Reliability was confirmed with a Cronbach’s alpha of 0.78^30^; *(iii) Self-Care of Heart Failure Index (SCHFI)*: The questionnaire developed by Riegel *et al.*, this questionnaire contains 15 questions across three subscales: Self-care maintenance (5 items), Self-care management (6 items), Self-care confidence (4 items). Responses are rated on 4- or 5-point Likert scales (scored from 0-4 or 1-4, depending on the item). Each subscale is standardized to a 100-point score, with higher scores indicating better self-care.^31^ The validity and reliability of the Persian version were assessed by Moadab *et al.*^32^ Content validity was evaluated by 12 faculty members from Guilan University of Medical Sciences, and after collecting feedback and applying necessary revisions, the final version was prepared. The content validity index for this questionnaire was above 0.83. Internal consistency reliability was measured using Cronbach’s alpha, which was greater than 0.80.

Ethical Considerations. This study was approved by the Ethics Committee of Jahrom University of Medical Sciences under the approval number IR.JUMS.REC.1402.054. Participants were fully informed about the study procedures, duration, confidentiality, and anonymity of their data. Participation was voluntary, and both verbal and written informed consent were obtained.

Statistical Analysis. Data were analyzed using SPSS version 22, with a significance level of 0.05. Descriptive statistics (mean, standard deviation, frequency, and percentage) and inferential statistics with a 95% confidence level were applied. The Shapiro-Wilk test was used to assess the normality of variable distribution. Within-group comparisons were performed using the Paired sample t-test and Wilcoxon test. Between-group comparisons were conducted using the Independent Sample t-test and Mann-Whitney U test.

Trial Registration Code and Date: IRCT20210114050037N2 in the Clinical Trial Registration Center of Iran (Registration date: 02/09/2023). 

## Results

Baseline demographic characteristics were comparable between the two groups. The mean age was 60.26 ± 10.52 years in the intervention group and 61.03 ± 10.72 years in the control group (*p*=0.781). The proportion of male participants was similar in both groups (*p*=0.85). No significant differences were observed in other baseline variables (Table 1).

**Table 1:** Comparison of Intervention and Control Groups Based on Demographic Variables

Variables		Groups		***p*-value**
		Intervention	Control	
n (%)	n (%)
Sex	Male	20 (66.7)	16 (53.3)	0.125*
	Female	10 (33.3)	14 (46.7)	
Education	High school	27 (90.0)	25 (83.3)	0.737**
	Diploma	2 (6.7)	3 (10.0)	
	University education	1 (3.3)	2 (6.7)	
Job	Freelance	18 (60.0)	12 (40.0)	0.482**
	employee	2 (6.7)	3 (10.0)	
	Housekeeper	9 (30.0)	13 (43.3)	
	Unemployed	1 (3.3)	2 (6.7)	
Marital status	Married	26 (86.7)	24 (80.0)	0.365*
	Single	4 (13.3)	6 (20.0)	
Residence	Urban	21 (70.0)	21 (70.0)	
	Rural	9 (30.0)	9 (30.0)	0.611*
History of hospitalization	Once	5 (16.7)	5 (16.7)	0.309**
	Twice	6 (20.0)	11 (36.7)	
	More than twice	19 (63.3)	13 (43.3)	
	Never	0 (0)	1 (3.3)	
Underlying disease	Yes	25 (83.3)	23 (76.7)	
	No	5 (16.7)	7 (23.3)	0.374*
Smoking	Yes	1 (3.3)	4 (13.3)	
	No	29 (96.7)	26 (86.7)	0.177*

*Fisher’s exact test, **Chi-square, ***Independent t-test

The findings indicated that the overall SCHFI had a normal distribution before and after the intervention in both the intervention and control groups; therefore, parametric tests were used. However, the subscales of the SCHFI did not follow a normal distribution. The HFHLQ and its subscales did not follow a normal distribution before and after the intervention in both groups; thus, non-parametric tests were applied. In Within-Group Comparison Wilcoxon test results showed a significant increase in the mean health literacy score and its subscales in the intervention group after the intervention compared to before (*p*<0.001). Likewise, the mean self-care score and its subscales significantly increased in the intervention group after the intervention (*p*<0.001). In Between-Group Comparison Mann-Whitney test results revealed no significant statistical differences between the intervention and control groups in terms of health literacy, its subscales, self-care, and its subscales before the intervention. However, after the intervention, a significant difference was observed between the two groups in the mean scores (*p*<0.05), with the intervention group showing higher scores than the control group (Table 2).

**Table 2:** Comparison of the mean score of health literacy and self-care in the intervention and control group, before and after the intervention

Variables	Moment	Groups	***p-*value (between- groups)**	t/z
		Intervention			Control
Health literacy	Before	31.03 ±5.40	31.20±6.06	0.810*	0.241
After	41.54±7.56	31.33±6.14	0.001*	-4.493
*p-*value (within groups)***	<0.001	0.257		
Z	4.221	1.134		
Functional health literacy	Before	9.87±2.52	10.63±3.75	0.343*	0.948
	After	12.89±3.76	10.63±3.75	0.027*	-2.217
*p-*value (within groups)***	0.001	0.999		
Z	3.480	-		
Communicative health literacy	Before	10.77±2.03	10.93±2.78	0.724*	0.353
	After	14.50±2.33	11.03±2.77	<0.001*	-4.509
*p-*value (within groups)***	<0.001	0.083		
Z	4.127	1.732		
Critical health literacy	Before	10.40±1.79	9.63±2.41	0.185*	-1.327
	After	14.14±2.40	9.67±2.41	<0.001*	-5.443
*p-*value (within groups)***	<0.001	0.655		
Z	4.230	0447		
Self-care	Before	51.13±8.97	50.43±12.62	0.609**	-0.512
	After	79.41±13.69	50.28±12.66	<0.001**	-5.601
*p-*value (within groups)****	<0.001	0.662		
t	4.288	-0447		
self-care maintainance	Before	36.89±13.98	37.11±15.82	0.905**	-0.120
	After	61.43±18.62	37.11±15.82	<0.001**	-4.617
*p-*value (within groups)****	<0.001	0.999		
t	4.04	-		
Self-care management	Before	56.83±12.49	58.00±14.60	0.922**	0.098
	After	88.04±15.30	57.67±14.78	<0.001**	-5.343
*p-*value (within groups)****	<0.001	0.655		
t	4.291	-0447		
Self-care confidence	Before	59.44±13.44	54.44±20.73	0.384**	-0.871
	After	87.50±15.63	54.44±20.73	<0.001**	-5.063
*p-*value (within groups)****	<0.001*	0.999		
t	4.131	-		

*Mann Whitney U tests, **Independent t-test, ***Wilxacon, ****Paired t test

## Discussion

This study aimed to determine the effect of family-centered self-care education via mobile phone education on health literacy and self-care in patients with heart failure. The results showed that family-centered education through smartphones led to an improvement in health literacy and self-care status in the intervention group.

In the present study, the target group for education consisted of active family members of patients, and virtual education for them successfully increased health literacy and self-care behaviors in patients. Consistent with the findings of this study, Barkhordari *et al.* investigated the impact of improving health literacy through virtual education in patients with heart failure and found that virtual education led to enhanced health literacy in these patients. However, in their study, the Ispring Play software was used for education, which differs from the software utilized in the present study, though the variable under investigation and the educational approach remained the same.^25^ In the study by Khajavi *et al.*, which examined the effect of a web-based family-centered supportive educational program on adherence to the treatment regimen in heart failure patients after discharge, it was also found that this program had positive effects on treatment adherence. In their study, educational content was sent to patients and their families via the Soroush application, and weekly follow-ups were conducted by researchers through phone calls. This study aligns with the present research in terms of implementing family-centered education virtually and its positive impact.^33^ In the study by Huang *et al.*, it was also found that the use of a smart application installed on patients’ mobile phones had a significant impact on the self-care of patients with lung cancer.^34^

Dunbar *et al.* examined the impact of family involvement and educational interventions on heart failure. In this study, heart failure patients and one of their family members were included and divided into three groups. The first group received standard care, the second group participated in group education sessions, and the third group, in addition to attending an educational session, received support via the internet and email. At the end of the study, at both 4-month and 8-month follow-ups, the third group demonstrated better dietary adherence compared to the other two groups.^35^ In line with these findings, the study by Jafari *et al.*, which conducted an eight-session, two-hour, in-person family-centered empowerment training for patients with heart failure and their primary caregivers, found that this model can improve daily living activities in elderly patients with heart failure. Although the type of training and the variables examined in their study differ from those in the present research, both studies are consistent in highlighting the positive impact of family-centered education on patients with heart failure.^36^

Similarly, in the study conducted by Srisuk *et al.*, which aimed to determine the effect of a family-centered educational program on the level of awareness, quality of life, self-care behaviors, and perceived control of caregivers in managing the symptoms of patients with heart failure, it was found that patients and caregivers who participated in the family-centered educational program achieved higher average scores in all examined variables compared to the control group at 3 and 6 months after the intervention. This difference was statistically significant.^37^ In the study conducted by Pashaei *et al.* on the effect of family-centered educational support on the quality of life of patients with permanent cardiac pacemakers, it was found that implementing family-centered educational support was effective in improving the quality of life of these patients. The educational intervention in this study was conducted in four in-person sessions, attended by both the patient and an active family member.^38^

Studies have been conducted on the impact of family-centered education on various diseases. Among them, the study by Katebi *et al.* compared the effects of family-centered education with individual-centered education on the quality of life of patients with type 2 diabetes. The results showed that family-centered education, similar to individual-centered education, can effectively improve the quality of life of diabetic patients ^18^. Additionally, in the study by Kiani *et al.* on the effect of the family-centered empowerment model on the resilience of family caregivers of patients undergoing hemodialysis, it was found that this empowerment model enhances the resilience of caregivers.^39^

Studies have also been conducted on the implementation of self-care education through both in-person and virtual methods for patients themselves, some of which will be examined in relation to the present research. For example, in the study by Eghtedar *et al.*, positive effects were observed in terms of treatment adherence and hospital readmission rates among heart failure patients who received self-care education via smartphone.^40^ Additionally, in the study by Ahmadi *et al.*, it was found that virtual education had a positive impact on self-care and quality of life in patients with heart failure.^41^ Similarly, the study by Khezerlou *et al.* demonstrated that virtual education positively influenced self-care behaviors in heart failure patients.^42^These studies align with the present research regarding the positive effects of virtual education on heart failure patients. However, while the mentioned studies provided virtual education directly to the patients, the present study implemented virtual education in a family-centered manner.

In the study by Sharifzadeh *et al.*, which examined the effect of virtual self-care education related to COVID-19 on the life expectancy of pregnant women-delivered via WhatsApp-it was found that virtual self-care education did not significantly impact their life expectancy. The results of this study do not align with the present research, which could be attributed to differences in the target group and the messaging platform used for education.^43^ Similarly, in the study by Badiyepeyma *et al.* on the effect of remote self-care education on functional status and quality of life in patients with rheumatoid arthritis, it was found that distance education alone did not have a significant impact on these patients. The results of this study also do not align with the present research, which may be due to differences in the target group and the educational content.^44^

In the study by Hsu *et al.*, which examined the impact of a self-regulation program on self-care behaviors in heart failure patients, it was found that self-regulation programs can effectively improve self-care behaviors in these patients.^45^ The implementation method in this study differs from the present research, as the education was provided in-person and directly to the patients. However, the examined variable is the same, and the intervention ultimately led to an increase in self-care behaviors in these patients. Similarly, in the study by Kobraei *et al.*, it was found that in-person education can enhance health literacy in heart failure patients. This study included five in-person educational sessions covering physical activity, diet, treatment recommendations, follow-up, and medication adherence for the patients themselves. Although the intervention method was in-person and directed at the patients, the educational content was similar to that of the present study, leading to an improvement in health literacy. Therefore, from this perspective, the findings align with the present research.^27^

The most significant strength of the present study was the use of mobile phone for education, which offered several advantages. First, patients received essential health recommendations without wasting time or incurring costs, indirectly reducing economic burden, improving accessibility, and increasing convenience. Second, the ability to send educational files in video and audio formats made this platform more engaging and acceptable for patients and their families compared to SMS. Third, heart failure patients and their families could potentially share the educational video in their family and social group, influencing the lifestyle and disease management practices of a broader audience. 

This study has several limitations. First, the sample size was relatively small, and the sampling period was extended, which may affect the representativeness of the findings. Second, the study was conducted at a single center, limiting the generalizability of the results to other patient populations. Third, there was no long-term follow-up to assess the sustainability of the intervention’s effects. Future studies are recommended to include longer follow-up periods to better evaluate the lasting impact of this educational method. Another limitation is the lack of blinding during data collection, as both the researchers and participants were aware of group allocation. This was due to the nature of the intervention and the single-center design. Additionally, the outcome assessor was the investigator, which may introduce potential bias. However, to mitigate this risk, all data were collected using validated questionnaires through structured telephone interviews, following standardized instructions for all participants. Moreover, the data analyst was blinded to group allocation, which helped maintain objectivity in the statistical analysis.

Conclusion. The findings demonstrated that implementing a mobile-based, family-centered self-care education program increased health literacy and self-care behaviors in heart failure patients. Therefore, it is recommended that mobile phone education be integrated into routine care for heart failure patients, particularly for those who cannot attend in-person educational sessions due to physical limitations, distance, cost, or other barriers. Additionally, leveraging the active participation of family members in patient education can contribute to improving patients’ overall health outcomes. It is recommended that future research explore mobile phone using other virtual education platforms with larger sample sizes and over longer durations follow up to better determine its long-term effectiveness.

## Availability of data and materials

The data that support the findings of this study will be available from the corresponding author upon reasonable request.
